# Comparative Analysis of the Chloroplast Genomes of *Grewia tembensis* Fresen and Closely Related Species of *Grewioideae* Hochr: A Phylogenetic and Conservation Perspective

**DOI:** 10.3390/genes16101124

**Published:** 2025-09-23

**Authors:** Widad S. AL-Juhani

**Affiliations:** Department of Biology, Faculty of Science, Umm Al-Qura University, Makkah 24381, Saudi Arabia; wsjuhani@uqu.edu.sa

**Keywords:** *Grewia tembensis*, chloroplast genomes, comparative analysis, phylogenetic relationships, SSC, LSC, RSCU, SSRs

## Abstract

Background: *Grewia* is a genus of flowering plants belonging to the *Malvaceae* family. *Grewia tembensis* is used in traditional medicine for the treatment of several microbial diseases as well as a livestock feed. Methods: In the current study, the complete chloroplast (cp) genome of *G. tembensis* was constructed using data derived from high-throughput sequencing, followed by comprehensive analyses and comparison with phylogenetically related species. Results: The chloroplast genome of *G. tembensis* is 158,040 bp long and has the typical quadripartite structure found in angiosperms. The large single-copy (LSC) segment measures 86,956 bp, whereas the small single-copy (SSC) regions encompass 20,142 bp. The two inverted repeat (IRa and IRb) regions have an identical length of 25,471 bp and display a higher degree of conservation relative to the single-copy (SC) regions based on nucleotide diversity analysis. The genome of *G. tembensis* possesses 130 genes. The simple sequence repeat (SSR) numbers ranged between 202 and 234 repeats in *Grewioideae* subfamily species under this study. Furthermore, nucleotide diversity analysis demonstrated a marked elevation in polymorphism information (Pi) values across 30 genes in *Grewioideae*. Conclusions: cpSSRs can be used for the examination of population genetic variability within and between *Grewia* species, as well as the categorization of populations and their biogeographical distribution. In addition, loci with high Pi values can contribute substantial genetic variability, which is crucial for addressing taxonomic dilemmas in phylogenetic investigations.

## 1. Introduction

The *Malvaceae* s.l. family includes controversial taxa, with many genera poorly defined and lacking monophyletic testing [1]. *Malvaceae* includes nine subfamilies: *Bombacoideae* (Burnett), *Brownlowioideae* (Burret), *Byttnerioideae* (Burnett), *Dombeyoideae* (Beilschm.), *Grewioideae* (Hochr.), *Helicteroideae* (Meisn.), *Malvoideae* (Burnett), *Sterculioideae* (Beilschm.), and *Tilioideae* (Arnott) [2]. *Grewia* is a genus of flowering plants belonging to the *Malvaceae* family and the *Grewioideae* subfamily. To date, 274 accepted species have been identified within the *Grewia* genus [3]. The *Grewia* L. genus was first classified in the *Tiliaceae* family. However, subsequent phylogenetic analysis has reassigned it to the *Malvaceae* family, and *Tiliaceae* has become a subfamily of *Malvaceae* (Tilioideae).

*Grewia tembensis* is a shrub that is endemic to desert or desert shrubland habitats, with a geographical distribution including Algeria, Eritrea, Ethiopia, Saudi Arabia, Somalia, Sudan, and Yemen. However, *G. tembensis* appears to be uncommon in the mountainous areas of Yemen, with reports only describing a single case [4].

*Grewia tembensis* is used in traditional medicine for the treatment of cough, and its branches are used as tools for dental hygiene and as a source of firewood [5]. *Grewia tembensis* is used for the treatment of microbial infections in Djibouti, such as abscesses and furuncles, and its fruits are eaten. The Kenyan Maasai population employs *G. tembensis* alongside ovine droppings to heal cracks in the cranium, whereas its roots are used to treat typhoid, heartburn, hypochondria, anorexia, and diaphragm swelling. *Grewia tembensis* is similarly used in the treatment of breast cancer in Tanzania. Farmers also use *G. tembensis* for livestock forage and to create fences around their homes [6].

**Figure 1:**
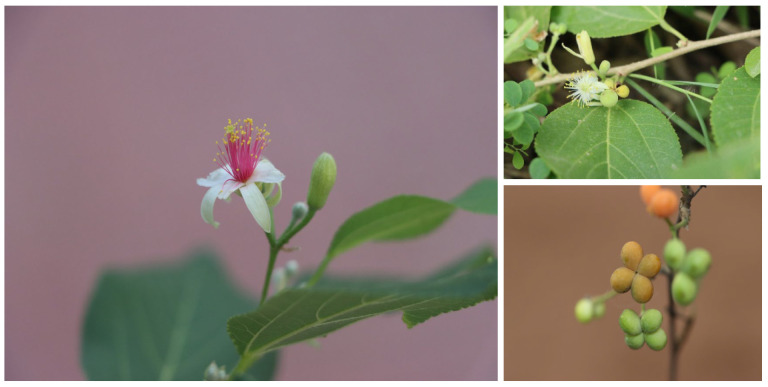
*Grewiatembensis* naturally grown in the Fayfa Mountains of southwestern Saudi Arabia.

Mature *G. tembensis* plants typically reach a height of up to 4 m and have long, thin, and smooth gray stems. Its leaves are simple and 3–4 cm long, obovate to elliptic with a rounded apex, serrated margins, and a rounded base. The surface of *Grewia tembensis* leaves is green above and greenish gray below. In addition, its upper stems are typically covered with hairs with woolly trichomes. The flowers are borne in inflorescences and include five sepals on the calyx and five petals on the corolla, which are white with a pink base. The flowers feature many free stamens. The 4–6 mm drupe pericarp contains 2–4 lobes, with the fruits being green when unripe and orange upon ripening [4,5], Figure 1. Two infraspecific taxa have been reported and recognized within *Grewia tembensis*: *Grewia tembensis* var*. Burret* and *Grewia tembensis* var*. Tembensis* [3].

Aljuhani [7] conducted a comparative study using SEM to examine *G. tembensis* and *G. trichocarpa* from the Fifa region in southern Saudi Arabia. The study identified distinct micromorphological features that differentiate the species, particularly in epidermal structure, stomatal characteristics, and trichome traits.

In terms of molecular phylogenetic studies, Dorr and Wurdack [1] recently reconstructed a phylogenetic tree of the genus *Grewia* using ITS sequence data, which includes a number of species from other taxa in the sub-family *Grewioideae*. The links that were observed for *Grewia* suggest considerable biogeographical complexity. However, the authors noted that these results could be affected by the relatively small number of taxa and genetic sequences included in their analysis. The investigators argued that plastome sequences should be used, due to their high conservation rates [8].

Plastids represent specialized organelles located within plant cells, functioning as crucial locales for various vital biological processes, including photosynthesis. The evolutionary origin of plastids is linked to a primordial endosymbiotic occurrence, which has led to modern plastids possessing a genome that is markedly diminished relative to their independently living ancestors [9,10]. Typically, the plastome exhibits a conserved quadripartite circular genomic structure consisting of two inverted repeat (IR) regions alongside two single-copy (SC) regions, designated as the large single-copy (LSC) and small single-copy (SSC) regions. Nevertheless, certain species, such as Medicago (Fabaceae), have experienced a loss of one of the inverted repeat regions [9].

With the advent of sophisticated high-throughput DNA sequencing methodologies, a vast array of plastid genomes has been made accessible in public databases. Chloroplast genomes have become pivotal in the investigation of molecular evolution and phylogenetic research, attributable to their favorable size for sequencing and the remarkable collinearity identified among chloroplast genomes across diverse plant taxa, thereby facilitating comparative studies. Plastid phylogenomics has demonstrated efficacy in clarifying phylogenetic interrelations among species, as well as in analyzing population genetic frameworks within terrestrial plants, including green algae [11].

Recent advancements in genomic research techniques have propelled the emergence of contemporary applications, such as the creation of pan-plastomes, which aid in delineating all nucleotide variants existing within a lineage through rigorous sampling and comparative analyses. Such datasets can yield comprehensive insights into the maternal lineage of a species and enhance the understanding of practical aspects such as domestication history or asymmetries in maternal inheritance, thereby informing future breeding initiatives, facilitates the exploration of diversity and population structure relationships within the genus while revealing genetic variations and potential molecular marker loci within the plastome [9]. For instance, numerous pan-plastomes have been established for agriculturally significant species, such as a comprehensive map of *Gossypium* genus, the pan-plastome of *Hemerocallis citrina*, as well as those of *Prunus mume* and *Pea* plastomes [9,12,13,14].

Numerous chloroplast genomes have been documented within the plant family *Malvaceae*, with notable studies including investigation of the cp genome of *Hibiscus taiwanensis* [15]. Additionally, Li et al. [16] explored the chloroplast genome of *Malva verticillata*. The examination of the plastome of *Hibiscus syriacus* L. Mamonde was conducted by Kim et al. [17]. Furthermore, the chloroplast genome of *Hibiscus cannabinus* was sequenced by Cheng et al. [18]. Wu et al. [19] analyzed the complete chloroplast genome of *Diplodiscus trichospermus*, which belongs to the *Brownlowioideae* subfamily. Moreover, a study was conducted on the chloroplast genomes of six species within the *Malvaceae* family [20]. The characterization and phylogenetic analysis of *Theobroma bicolor* genomes originating from Peru were performed by Tineo et al. [21]. Additionally, the complete chloroplast genome of *Durio zibethinus* L. cultivar Ri6 (*Helicteroideae*, *Malvaceae*) was examined by Huy et al. [22].

Nevertheless, research on *Grewia* chloroplasts is limited. For example, Xu et al. [23] reported the complete chloroplast genome of *Grewia biloba*, whereas Hou et al. [24] reported the complete chloroplast genome of *Grewia biloba* var*. Parviflora*. In addition, these studies did not perform comparative studies between the different species and genera that belong to the *Grewioideae* subfamily. Similarly, only two species of *Grewioideae* were referenced for analysis of their plastomes and phylogenetic affiliations in a recent study conducted by Wang et al. [25].

Thus, this work aimed to construct the complete chloroplast (cp) genome of *G. tembensis* and perform a comparative analysis of the species in the genus *Grewia* L. In addition, this study sought to construct a phylogenetic tree of related taxa in the subtribe *Grewioideae*, verify the taxonomic status of the species *G. tembensis*, and examine the evolutionary relationships within the subtribe.

## 2. Materials and Methods

### 2.1. Sample Acquisition

*Grewiatembensis* was collected from the Fayfa Mountains, which are located in the Jazan region of southwestern Saudi Arabia (17°14′45.1″ N 43°05′27.2″ E). This region in the southwestern area of the Kingdom of Saudi Arabia is a crucial zone for flowering plant species and flora, having multiple habitats and representing a unique biodiversity hotspot of the Arabian Peninsula. These features can be attributed to sufficient annual precipitation (above 300 mm) and a strong altitudinal gradient (from sea level to 3100 m). In addition, this southwestern region of Saudi Arabia has significant botanical and phytogeographic value, serving as a biogeographical corridor between the continents of Asia and Africa [26].

The *Grewia tembensis* samples were collected in November 2024 and taxonomically identified using a herbarium collection and appropriate literature. The exemplary samples were prepared and subsequently archived in the Umm Al-Qura University herbarium in the Makkah region, Saudi Arabia. To facilitate DNA extraction, fresh foliar samples were dehydrated using silica gel.

### 2.2. DNA Extraction

The deoxyribonucleic acid (DNA) was extracted from dried leaves of *Grewia tembensis* using the CTAB DNA isolation technique, as described previously by Doyle and Doyle [27]. A N120 nanophotometer (Implen GmbH, München, Germany) was used to assess DNA purity based on the 260/280 and 260/230 absorbance ratios.

### 2.3. Genomic Library Development

A library was constructed using an input of 1.0 µg of DNA for sample generation. The DNA library was prepared according to the guidelines of the Illumina TruSeq Nano DNA Kit, as described by the manufacturer’s protocol. Random fragmentation of the DNA samples yielded an average fragment size of approximately 350 bp. The size distribution of the template fragments was evaluated using an Agilent Technologies 2100 Bioanalyzer (Agilent, Santa Clara, CA, USA), and the libraries were quantified using qPCR.

### 2.4. Genome Sequencing

Clusters were generated by inserting the library into a flow cell, which aided with successful hybridization of the fragments to a substrate with surface-anchored oligonucleotides complementary to the adapter for the library. Next, all the fragments were amplified into separate, clonal clusters using bridge amplification. After cluster generation, the generated templates were sequenced on the Illumina platform using the standard sequencing by synthesis (SBS) protocol. The raw sequencing data were processed with Trimmomatic v0. 38 [28] to remove adapter sequences and filtering reads against a quality threshold (Q30 at 91%) to yield a net output of 12 Gb of filtered reads. Macrogen (https://dna.macrogen.com/, accessed on23 May 2025) performed the library preparation and sequencing.

### 2.5. Genome Composition and Annotation

The raw reads were assessed using the FastQC software(v0.12.0). The filtered reads were subsequently used for genomic assembly with NOVOPlasty version 4.3.1 [29] based on a k-mer value of 33 for reconstruction of the full chloroplast genome from the *G. tembensis* total genomic sequence. Sequences of *Grewia chungii* (GenBank accession number: NC_054166), *Grewia biloba* (NC_058214.1), and *Grewia biloba* var*. parviflora* (ON882041. 1) were used as references during assembly.

The assemblies corresponded to full chloroplast genomes in a single contig. The cp genome of *G. tembensis* was predicted and annotated using the GeSeq tool [30] with the default parameters (thresholds were set to ≥60 and ≤85 for protein-coding genes and RNAs, respectively). tRNAscan-SE version 2.0 [31] was used to detect tRNA genes. Circular maps of the chloroplast genomes were produced using OGDRAW (Organellar Genome DRAW), version 1.3.1 [32], via the gb format of the annotated sequence files. The chloroplast genome of *G. tembensis* has been deposited in GenBank with the accession identifier PV096988.

### 2.6. Sequence Evaluation

Relative synonymous codon usage (RSCU) metrics, including nucleotide composition and the utilization of codons, were examined using Graphical Codon Usage Analyzer Version 1.2 (GCUA) [33]. Potential RNA editing loci in the protein-coding sequences were predicted using the PREPACT3 suite [34] with a threshold criterion of 0.8.

### 2.7. Chloroplast Genome Repeat Analysis

MIcroSAtellite (MISA) v2.0 [35] was used to identify simple sequence repeats (SSRs) in the *G. tembensis* cp genome and seven other members of the subfamily *Grewioideae*, including *Grewia chungii*, *Grewia biloba*, *Grewia biloba* var*. Parviflora*, *Microcos paniculata, Colona floribunda*, *Corchorus capsularis*, and *Corchorus olitorius*. For mononucleotides, dinucleotides, trinucleotides, tetranucleotides, pentanucleotides, and hexanucleotide SSR motifs, eight, five, and four repeat units were applied.

The REPuter [36] program was implemented with default parameters to identify palindromic, forward, reverse, and complement repeats in the *G. tembensis* plastid genome and seven species from the *Grewioideae* lineage.

### 2.8. Comparative Genome Examination

The *G. tembensis* chloroplast genome was compared with those of seven representative taxa of the *Grewioideae* subfamily, namely, *Grewia chungii*, *Grewia biloba*, *Grewia biloba* var. *Parviflora*, *Microcos paniculata*, *Colona floribunda*, *Corchorus capsularis*, and *Corchorus olitorius*. All sequences except those of *Grewia tembensis* were retrieved from the NCBI database. This analysis was cited in the *Grewia tembensis* annotation.

The annotated genomic sequence of *G. tembensis* served as a reference in the Shuffle-LAGAN configuration [37] to gain further information via the mVISTA web-based interface (https://genome.lbl.gov/vista/mvista/submit.shtml, accessed on 25 June 2025)[38]. Additionally, a comparison between the IR boundaries and the junctions of the SC regions was conducted using the IRscope tool [39].

### 2.9. Nucleotide Variability Values

Nucleotide diversity metrics (Pi) were derived from identical sequence alignment. Nucleotide variability was assessed with sliding window analysis executed in DnaSP v.6 11. 01, employing a window size of 600 bp and a step size of 200 bp [40]. The boundaries of the inverted repeats (IRs) and the junctions between the large single-copy (LSC) and small single-copy (SSC) regions were inferred based on GBseq annotations in conjunction with the IRscope tool [39] within the cp genomes of *Grewioideae* taxa, utilizing *Grewia tembensis* as a reference point.

### 2.10. Phylogenetic Assessment

Phylogenetic relationships were evaluated using chloroplast genomic sequences for members belonging to nine subfamilies of the *Malvaceae* family in GenBank. An outgroup for the phylogenetic analysis consisted of three species from the Malvales order (*Bixa rellana* (Bixaceae), *Dialyceras coriaceum* (*Sphaerosepalaceae*), and *Gonystylus affinis* (*Thymelaeaceae*)). The nomenclature of the species, familial classes, and GenBank accession codes are presented in Table S1.

The common genes among the selected organisms were extracted, and the number of single-copy orthologous genes was identified using the orthofinder tool [41,42], followed by concatenating the complete CDS. Sequence alignment was subsequently conducted using MAFFT v7.525 [43]. Phylogenetic trees were built using Mega [44] version 11.0 with maximum parsimony (MP) trees based on 100 bootstrap replicates.

The MAFFT-generated alignment file was used to perform a Bayesian inference analysis (BI) in MrBayes v. 3.2.6 [45], and the resulting BI trees were visualized with the ITOL tool [46].

## 3. Results

### 3.1. Grewia Tembensis Chloroplast Genome Features

**Figure 2:**
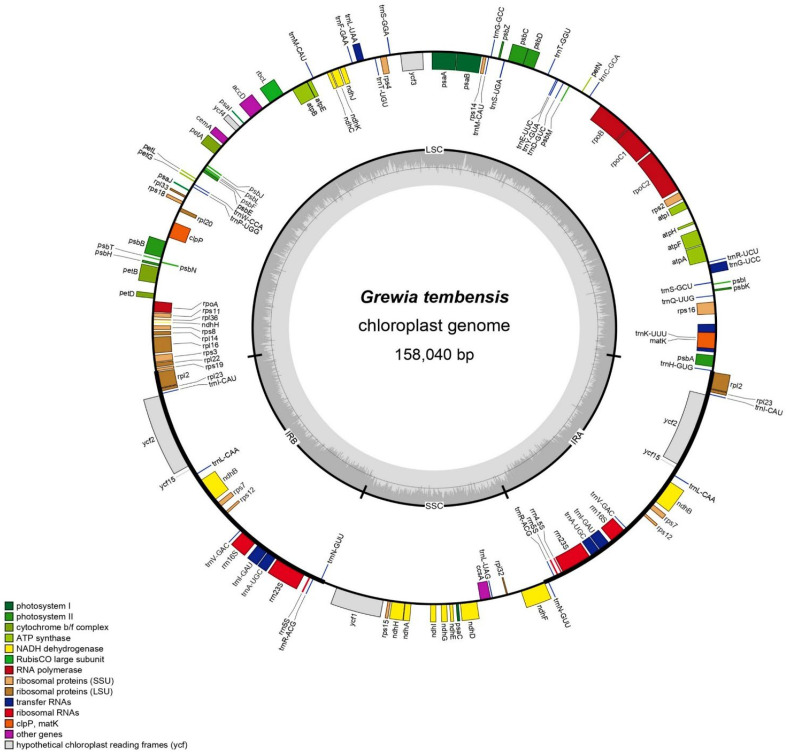
Genes of the chloroplast genome of *Grewia tembensis*. Genes situated externally to the circles are transcribed in a counterclockwise direction, whereas those located internally are transcribed in a clockwise direction. The functional genes are represented in the colored bar.

The chloroplast genome of *G. tembensis*, which is 158,040 bp in overall length (Figure 2), is circular. The chloroplast genome has a four-region structure, including large single-copy (LSC), small single-copy (SSC), and two inverted repeat (IRa and IRb) regions. The lengths of the LSC and SSC regions are 86,956 bp and 20,142 bp, respectively, whereas the lengths of the IRa and IRb regions are 25,471 bp each (Table 1).

The coding region covers 77,304 bp (47% of the genome), and the noncoding region covers 80,738 bp (51.08% of the genome). The adenine–thymine (AT) content accounts for 62.64% of the total genome, whereas the guanine–cytosine (GC) content accounts for 37.36%. The details of the genomic composition are described in Table 1, with A = 30.89%, T(U) = 31.76%, C = 18.85%, and G = 18.51% for *G. tembensis*. The functional annotation and gene classification of the *Grewia tembensis* chloroplast genome are presented in Supplementary Table S2.

A total of 130 genes were identified, including 110 unique genes. The chloroplast genome contains 79 protein-coding genes, 28 tRNA genes, and 3 rRNA genes (Table 1). A total of 60 protein-coding genes and 20 tRNA genes exist in the LSC region, 12 protein-coding genes and one tRNA are found in the SSC region, and 17 protein-coding genes and 14 tRNAs are found in the IR region. Most protein-coding genes start with a codon for methionine (AUG).

In total, 18 of the 130 genes of *Grewia tembensis* contained introns, including 11 protein-coding genes and 7 tRNA genes (Table 2). The ycf3 gene has two introns, whereas the remaining genes have only one. The LSC region has ten introns, whereas the IR regions have eight introns. Moreover, the trnK-UUU gene hosts the longest intron (2528 bp).

### 3.2. Relative Synonymous Codon Usage (RSCU)

**Figure 3:**
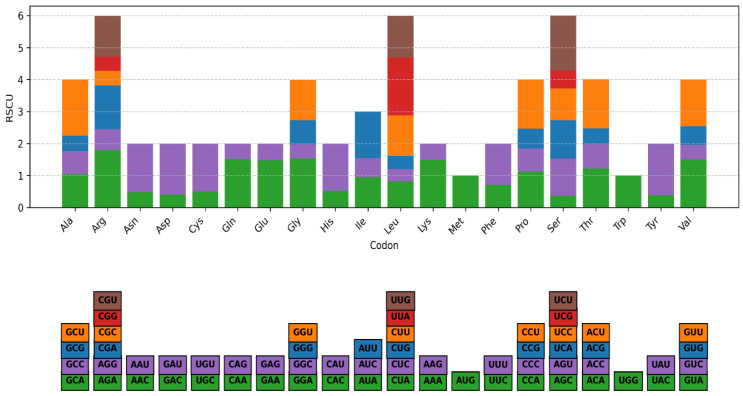
Amino acid composition of *G. tembensis* chloroplast protein-coding sequences. Colors refer to the different codons.

The composition of 20 amino acids across all protein-coding genes verified in the chloroplast genome of *Grewia tembensis* is represented in Figure 3. Codon usage bias in the plastome was evaluated using the nucleotide sequences of the protein-coding and tRNA genes. Analysis of protein-coding and tRNA genes (89,008 bp) from *Grewia tembensis* revealed the presence of 25,676 codons. As illustrated in Figure 3, leucine (10.37%) was the most abundant amino acid, whereas cysteine (1.16%) was the rarest. Codons encoding arginine, leucine, and serine are over-represented (higher than RSCU = 6), whereas tryptophan and methionine have the lowest abundance characterized by a lack of codon usage bias (RSCU value equal to 1). Based on the RSCU values provided in Table S3, 29 prefer codons with an RSCU value greater than 1, of which 28 end with either an A or U. In contrast, codons that end with a C or G have an RSCU value < 1 (Table S3), suggesting that the major amino acids prefer A/U-ending codons over C/G-ending codons.

### 3.3. RNA Editing Sites

Overall, 59 editing sites scattered throughout protein-coding genes were identified via genome editing. Most changes in the position of the codon were associated with the amino acids serine (S) and leucine (L) (from S to L); see Table S4. The results revealed the greatest number of editing sites—four each—in the rpoC2, rpoB, accD, and ndhD genes. In contrast, ycf1, rpl23, and matK genes each have three editing sites. In addition, atpA, rps2, rpoC1, rps14, rbcL, rpl20, and ndhF each have two editing sites, whereas atpF and ClpP each have only one editing site. Analysis of RNA editing revealed that some genes—such as accD, atpA, ndhA, ndhD, ndhE, petL, psaI, psbF, psbJ, psbN, psbT, rbcL, rpl20, rpl23, rpl36, rpoB, rpoC1, rps12, rps14, and rps2—do not have an anticipated locus at the initial codon of the foremost nucleotide.

### 3.4. Repeat Analysis

#### 3.4.1. Prolonged Repetitions

A total of 49 long repeats were observed in the *Grewia tembensis* chloroplast genome, including 20 palindromic, 10 forward, 18 reverse, and 1 complement repeats (Table S5). The protein-coding gene ycf2 is clearly represented, with two palindromic repeats and two forward repeats, giving it the highest number of repeats occurring in the *G. tembensis* cp genome.

**Figure 4:**
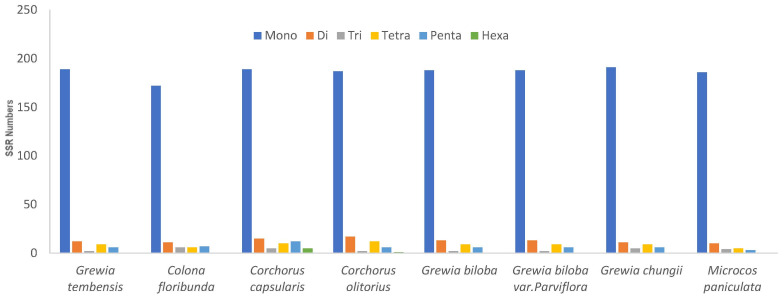
Number of repeat sequences found in eight *Grewioideae* plastomes (forward repeats are denoted by F, palindromic repeats by P, reverse repeats by R, and complement repeats by C).

Of the *Grewioideae* taxa, *Grewia biloba*, *Grewia biloba* var. *parviflora*, and *Corchorus capsularis* presented the highest frequency of palindromic repeats (21), whereas *Corchorus olitorius* had the highest frequency of forward repeats (35), as shown in Figure 4. Reverse repeats were not detected in any of the *Corchorus* species, whereas the reverse repeat frequency in *Grewia biloba* and *Grewia biloba* var*. Parviflora* was the highest (11). The greatest number of complement repeats was detected in *Colona floribunda* (3). Notably, complement repeats were absent in *Grewia biloba, Grewia biloba* var. *Parviflora, Corchorus capsularis,* and *Corchorus olitorius*.

#### 3.4.2. Simple Sequence Repeats (SSRs)

**Figure 5:**
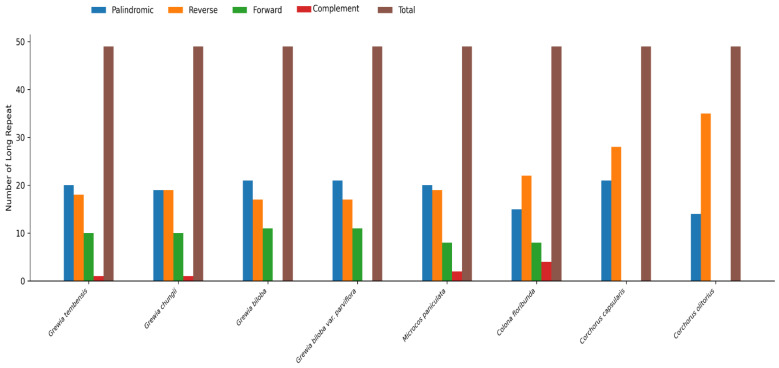
Enumeration of the various types of simple sequence repeats (SSRs) present within the chloroplast genomes of the eight *Grewioideae* species.

SSRs are widely distributed in chloroplast genomes. Across each of the eight cp genomes of the *Grewioideae* taxa, 202–234 SSRs were detected (Table S6). Single-nucleotide SSRs were the most common (172–191) and were composed mainly of A/T repeats. A repeats ranged from 70 to 101, and T repeats ranged from 80 to 94. In contrast, C and G repeats were less frequent. C repeats ranged from 4 to 7, and G repeats ranged from 1 to 4. Regarding the cp genome of *Grewia tembensis*, a total of 218 SSR markers were identified, of which 189 were mononucleotide repeats, mostly composed of A/T. Three types of dinucleotide SSRs were present in the *Grewioideae* species: AG/CT, AC/GT, and AT/AT. Two types of trinucleotides SSRs were present, namely, AAT/ATT and ATC/ATG. Tetranucleotide and pentanucleotide SSRs were the most diverse in the *Grewioideae* taxa. Nine tetranucleotide SSRs were noted, including AAAG/CTTT, AAAT/ATTT, AAGT/ACTT, AATT/AATT, AATC/ATTG, AAAC/GTTT, AGAT/ATCT, AATG/ATTC, and AACT/AGTT. Similarly, there were nine types of pentanucleotide SSRs (AAAAT/ATTTT, AATAT/ATATT, AAAGT/ACTTT, AACAC/GTGTT, AAAAG/CTTTT, AAGAT/ATCTT, AACAT/ATGTT, AAATT/AATTT, and AAACT/AGTTT) as shown in Table S6. In contrast, hexanucleotide SSRs were the least common in *Grewioideae* species. Four hexanucleotide types were identified exclusively in *Corchorus* species, including AATCAG/ATTCTG, AAAAAG/CTTTTT, AAAAAT/ATTTTT, and AAATAT/ATATTT, as shown in Figure 5.

Most of the SSRs were distributed in the LSC region, followed by the SSC region. Relatively few SSRs were located in the IR regions, as shown in Figure S1. With respect to the cp genome of *Grewia tembensis*, the majority of SSRs (63.3%) in the plastome were located in the LSC region, followed by 21.1% in the SSC region, while a smaller proportion (7.8% each) were located in the IRA and IRB regions.

### 3.5. Comparison of Genomes

**Figure 6:**
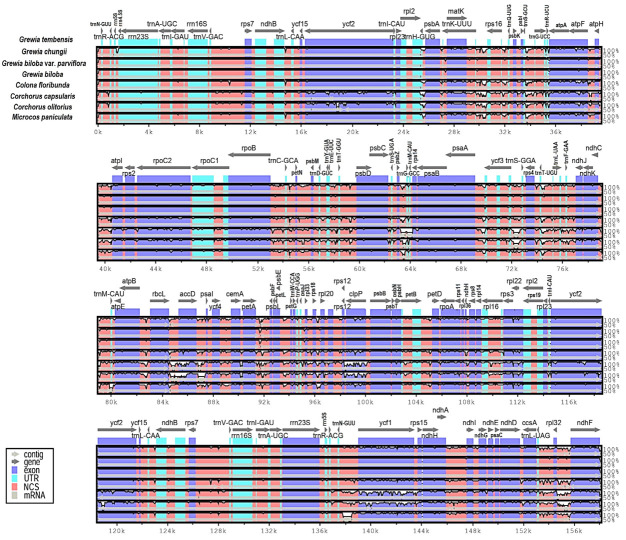
mVISTA plot of the complete chloroplast genome sequences and their genomic rearrangements for eight *Grewioideae* species. *Grewia tembensis* was used as the reference annotation. The *x*-axis indicates the coordinates in the chloroplast genome, and the *y*-axis represents the percentage identity from 50 to 100%. The gray arrows at the top indicate the location and orientation of each gene. The pink area displays the noncoding sequences (NCSs) in the annotation. The blue regions represent protein-coding genes, and the light-green area represents tRNAs and rRNAs.

To explore the level of dissimilarity in genome sequences at the whole-genome level, the mVISTA program was employed to analyze alignment sequences of *Grewia tembensis* with seven chloroplast genomes from the *Grewioideae* subfamily that are currently available in GenBank, including *Grewia chungii*, *Grewia biloba*, *Grewia biloba* var*. Parviflora*, *Microcos paniculata*, *Colona floribunda*, *Corchorus capsularis*, and *Corchorus olitorius*. In summary, protein-coding genes were more conserved than the noncoding genes, particularly the ndh, clpP, ycf2, trnH-psbA, rbcL-accD, ndhF, ycf1, accD, rps16, rps16-trnQ, and petA-psbJ genes (Figure 6).

Table S7 in the Supplementary Materials displays detailed information about coding and noncoding regions which showed a high level of variance in chloroplast genomes of the Grewioideae subfamily.

### 3.6. LSC/SSC and IR Boundaries

**Figure 7:**
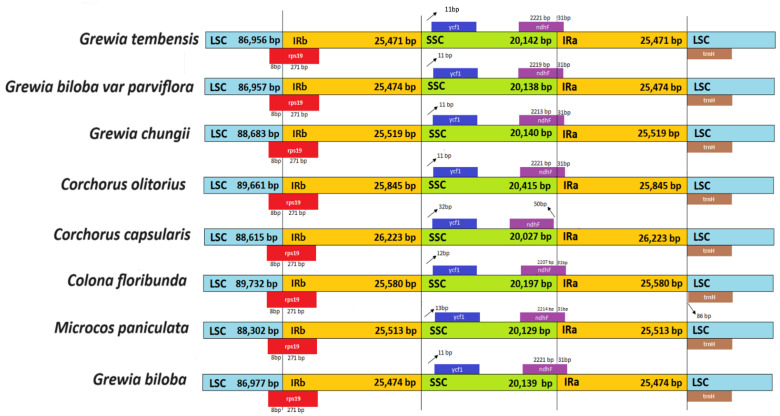
Comparison of the borders of the LSC, SSC, and IR regions in eight chloroplast genomes from the *Grewioideae* subfamily.

A comparative study of the LSC, SSC, and IR boundaries in the eight species of the subfamily *Grewioideae* is presented in Figure 7. LSC region lengths were in the range of 86,956–89,661 bp. The largest LSC region was noted in the species *Corchorus olitorius* at 89,661 bp, whereas the shortest was noted in *Grewia tembensis* at 86,956 bp.

Regarding the SSC regions in the *Grewioideae* species assessed in the present study, all eight available sequences exhibited similar lengths with few variations in the range of 20,027–20,415 bp. The IRa/IRb regions were also similar in most study species in the range from 25,471 to 25,845 bp, except *Corchorus capsularis* species, which contained the longest IR region among the studied species at 26,223 bp.

The boundaries of the LSC, SSC, and IR regions are occupied by the rps19, ndhF, and trnH genes, with few differences noted. The trnH gene was at the IRa–LSC boundary in all species. A small exception was observed in *Colona floribunda*. Here, the trnH gene was 86 bp away from the border (Figure 7). Another anomaly was the ndhF gene located in the SSC–IRa boundary in all species; however, in *C. capsularis*, the ndhF gene extended into the SSC region and 50 bp away from the SSC–IRa boundary.

Additionally, the ycf1 gene is located in the SSC region, with 11 bp in the IRb–SSC junction in *Grewia tembensis, Grewia chungii, Grewia biloba, Grewia biloba* var*. Parviflora,* and *Cochorus olitorius*. In contrast, the ycf1 gene is located 12 bp, 13 bp, and 32 bp away from the IRb–SSC junction in *Colona floribunda*, *Microcos paniculata*, and *Corchorus capsularis*, respectively. The rps19 gene occupied the LSC–IRb boundaries in all species.

Overall, the IR boundaries of these *Grewioideae* species were highly conserved, with only minor differences observed in gene locations.

### 3.7. Nucleotide Variability

**Figure 8:**
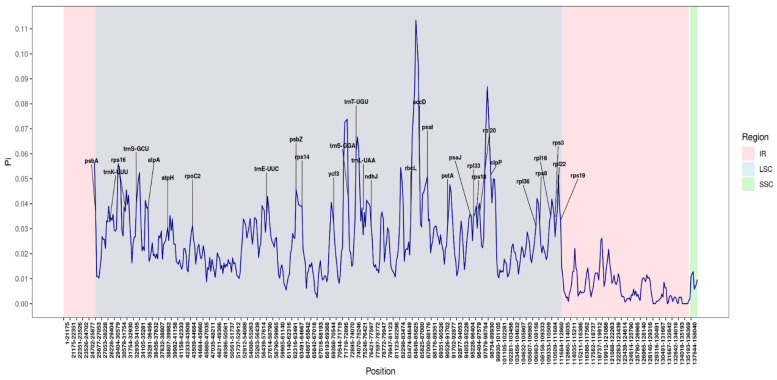
Sliding window analysis of the complete chloroplast genomes of eight species of the *Grewioideae* subfamily. Nucleotide variability was assessed using DnaSP with a window length of 600 bp and a step size of 200 bp. The *x*-axis represents the specific regions of the chloroplast genome, and the *y*-axis represents the nucleotide diversity for the corresponding window. The figure shows the 30 hypervariable regions with a pi value greater than 0.03.

Variable regions identified with pi ≥ 0.03 are shown in Figure 8, which presents the nucleotide diversity results. The diagram illustrates 30 nucleotide polymorphism sites distributed throughout the genome, serving as hotspots of variation, primarily within the LSC regions. The amount of polymorphic DNA, with values ranging from a Pi of 0.03048 to 0.06089 and a mean Pi of 0.038428, is shown in Table S8 for the genes for which pi values were obtained. Importantly, the analysis revealed that inverted repeats are more conserved than single-copy regions.

### 3.8. Phylogenetic Evaluation

**Figure 9:**
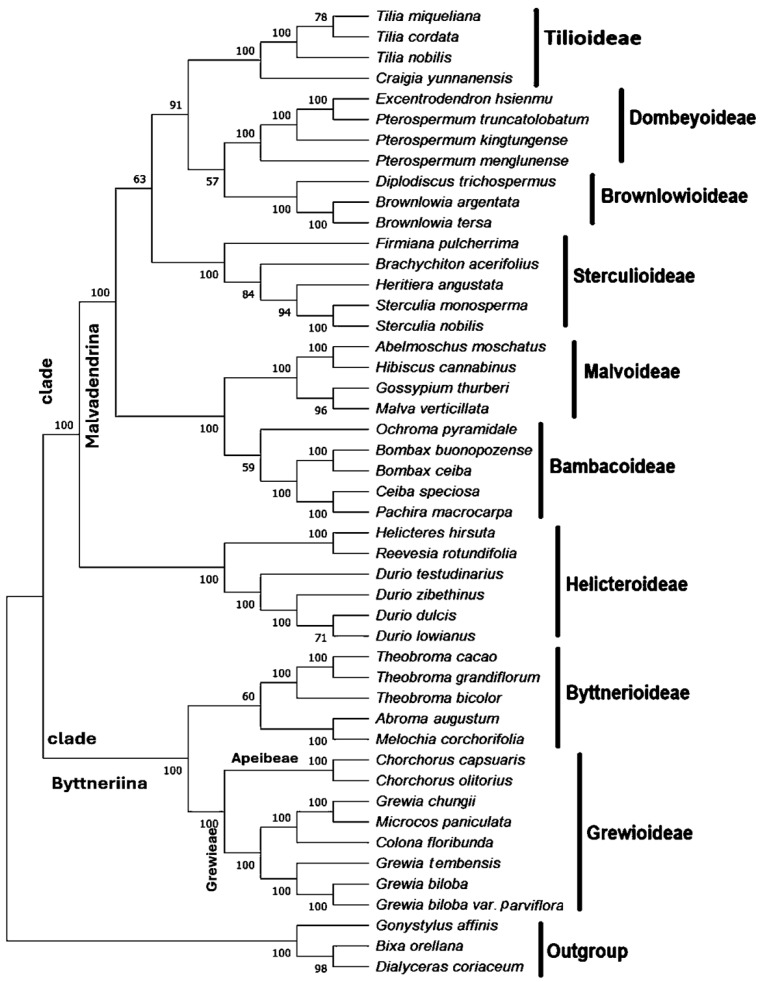
Phylogenetic trees constructed based on the coding sequences (CDSs) of different taxa using the maximum parsimony (MP) method. This tree depicts the phylogenetic relationships of nine *Malvaceae* subfamilies, in addition to the species within the *Grewioideae* subfamily. Bootstrap (BS) values are given at the branch nodes.

**Figure 10:**
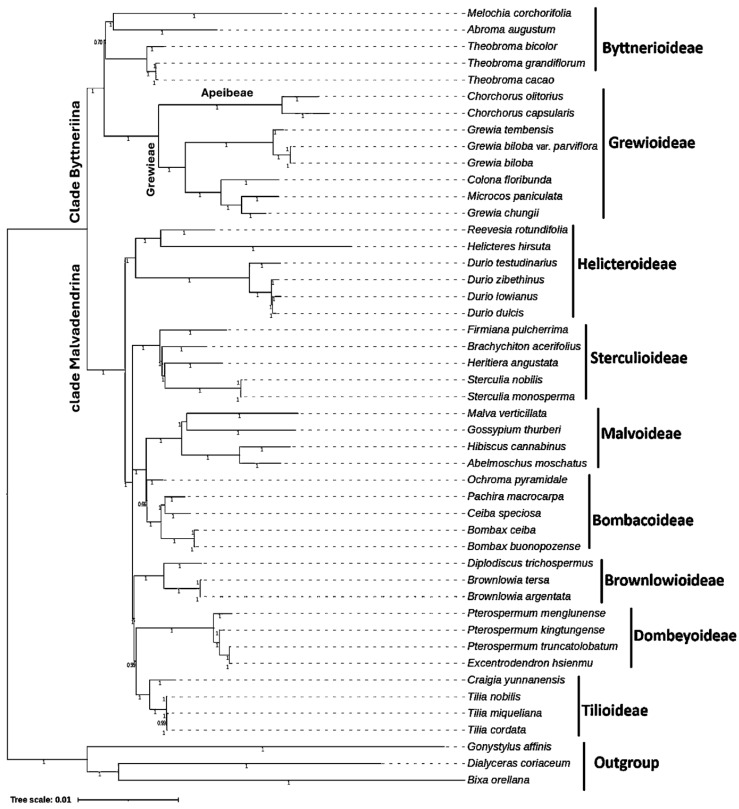
Phylogenetic trees constructed based on the coding sequences (CDSs) of different taxa using the Bayesian inference (BI) method. This tree depicts the phylogenetic relationships of nine *Malvaceae* subfamilies, in addition to species within the *Grewioideae* subfamily. Posterior probability (PP) values are given at the branch nodes.

Phylogenetic associations were analyzed using all presently accessible chloroplast genomes in *Grewioideae* (eight taxa) and representatives of all subfamilies of the family *Malvaceae* (*Bombacoideae, Brownlowioideae, Byttnerioideae, Dombeyoideae, Helicteroideae, Malvoideae,* and *Sterculioideae*). The outgroup representatives included three taxa from the order Malvales: *Bixa orellana* (Bixaceae), *Dialyceras coriaceum* (*Sphaerosepalaceae*), and *Gonystylus affinis* (*Thymelaeaceae*). The Bayesian inference (BI) and maximum parsimony (MP) approaches were used for phylogenetic analyses (Figure 9 and Figure 10). The derived phylogenetic trees showed topological congruence, with the majority of nodes achieving 100% bootstrap (BS) values and Bayesian posterior probabilities (PP = 1). This demonstrates that almost all nodes of the subfamilies represent highly supported monophyletic assemblages according to the results of this study.

The phylogenetic framework of *Malvaceae* is clearly divided into two major clades, Byttneriina and Malvadendrina, with the latter serving as the principal clade. In addition, Byttneriina also includes *Byttnerioideae* and *Grewioideae*. All branches in the *Grewioideae* subfamily were well supported (PP = 1; BS = 100%) and divided into two well-supported clades (PP = 1; BS = 100%). One clade contained members of the *Apeibeae* tribe (*Corchorus capsularis* and *Corchorus olitorius*), and the second clade contained members of the *Grewieae* tribe (including *Grewia tembensis, Grewia chungii, Grewia biloba and Grewia biloba* var. Parviflora*, Microcos paniculata,* and *Colona floribunda*). The location of *Grewia tembensis* was within the *Grewioideae* subfamily (Figure 9 and Figure 10). *Grewia tembensis* formed a clade with the *Grewieae* section with the highest bootstrap (BS) values (100%). All the members of the genus *Grewia* (*Grewia tembensis, Grewia biloba,* and *Grewia biloba* var*. parviflora*) cluster together, except *Grewia chungii*, which clustered with *Microcos paniculata*, forming a distinct clade with support values (PP = 1; BS = 100%).

On the other hand, the phylogenetic trees revealed strong phylogenetic affinity between *Malvoideae* and *Bombacoideae* as well as among the *Brownlowioideae*, *Tilioideae*, and *Dombeyoideae* subfamilies. *Sterculioideae* was the sister group of these groups, whereas *Helicteroideae* was located far away at the base of the *Malvadendrina* clade.

## 4. Discussion

The length of the chloroplast genome of *G. tembensis* (158,040 bp) resembled those of the chloroplast genomes of *Grewia biloba* and *Grewia biloba* var*. parviflora* (158,043 and 158,064 bp). This length was shorter than the cp genome lengths of the other species of the *Grewioideae* subfamily, including *M. paniculata* and *G. chungii* (159,450 and 159,861 bp, respectively), which were in turn shorter than those of *Colona floribunda, Corchorus capsularis,* and *Corchorus olitorius* (161,089, 161,088, and 161,766 bp, respectively). Overall, these *Grewioideae* species retained cp genome lengths that were consistent with the general range previously defined for the *Malvaceae* family. According to Wu et al. [19], the cp genome ranges from 157,936 to 168,953 bp among *Malvaceae* species, which is consistent with the cp genome size range of angiosperms (130,000–170,000 bp) [47]. These findings indicate the highly conserved size and structural integrity of the chloroplast genome in the *Malvaceae* family.

The chloroplast genomes of angiosperms are often characterized by a conserved configuration comprising two IR regions demarcating the SSC and LSC regions [19]. In conjunction with other angiosperms, *G. tembensis* has a conventional four-region architecture within its cp genome. The LSC size is 86,956 bp, and the SSC regions are 6038 bp and 20,142 bp, respectively. Moreover, the lengths of the two inverted repeat (IR) regions (IRa and IRb) are 25,471 bp, which are also within the conventional range of each region of the chloroplast genome in angiosperms, such as the LSC (80–90 kb), SSC (16–27 kb), and IR (20–28 kb) regions [25].

The *Grewia tembensis* chloroplast has a total GC content of 37.36. The GC content in the *Malvaceae* genera ranges from 35.83% to 37.34% [20], whereas the GC content is lower than the AT content. This finding is consistent with reports on other angiosperms with respect to their chloroplast genomes [19]. The chloroplast and mitochondria DNA is exclusively inherited through the maternal lineage [18], while the nuclear genome undergoes parental recombination during sexual reproduction rather than uniparental inheritance. As a result, the cp and mitochondrial genomes are more conserved than those of the nucleus [18].

The angiosperm-like chloroplast genome consists of 113 genes, including 79 protein-coding genes, 30 tRNA genes, and four rRNA genes [48]. The number of genes in the chloroplast genome of *G. tembensis* is similar to that observed in angiosperms. The genome contains 110 unique genes, including 79 protein-coding genes, 28 tRNA genes, and 3 rRNA genes. In addition, the results were consistent with the gene counts across several genera of the *Malvaceae* family, including the chloroplast genome of Kenaf (*Hibiscus cannabinus*) [18] and the complete chloroplast genome of *Diplodiscus trichosperma* [19]. Codons are fundamental for the translation of genetic information from mRNAs into proteins [49]. A preference in codon utilization significantly influences the evolutionary trajectories of chloroplast genomes, arising from selection and genetic alteration processes [50]. Codons encoding leucine were the most common, whereas those encoding cysteine were the least common in the plastome of *Grewia tembensis*. This observation has also been confirmed in many angiosperm chloroplast genomes [51]. Several studies have indicated that a high AT content is the major reason for the high prevalence of synonymous codons that terminate with A/U, which may be linked to natural selection and mutational occurrences throughout evolutionary processes [25,52].

In the cp genome of *Grewia tembensis*, the ycf3 gene is highly conserved with two introns, whereas the remaining genes harbor one intron. The sizes of the exons and introns of genes provide important information in research on chloroplast genomes. Introns are scattered across major taxonomic groups to interrupt genes [53]. These findings suggest that ycf3 genes could be targeted for studies of *Grewioideae* chloroplasts. On the other hand, exchanges at codon positions are mostly attractive for amino acid exchanges, such as serine (S) and leucine (L) (S to L), which have been similarly reported in different groups of angiosperms [54,55].

Simple sequence repeat (SSR) markers constitute powerful genetic markers for phylogenetic studies, population genetics measurements, and species identification given their high polymorphic nature and codominant inheritance [56]. This is especially important given the rapid and unprecedented progress in plant genomic research. So far, cpSSRs have made significant headway in studies related to plant population genetic diversity, analyses of population structure, classification of populations, and their biogeographic distributions.

For example, cpSSR markers were used tostudy the genetic diversity and population structure of ancient *Platycladus orientalis* L. (*Cupressaceae*) in China [57]. Similarly, new chloroplast SSRs were developed for *Pinus gerardiana* and applied in genetic diversity analyses [58]. Furthermore, chloroplast SSRs were employed to assess the genetic diversity and population genetic structure of *Paeonia suffruticosa,* an endemic shrub in China [59]. Additionally, genome-wide identification and development of SSR molecular markers were conducted for genetic diversity studies in medicinal species *Ilex asprella* [60]. In the present study, a total of 202 to 234 simple-sequence repeats (SSRs) have been identified within the chloroplast genome of *Grewioideae*. The chloroplast genomic data of *Grewia* species can be used to establish cpSSR markers that will be instrumental in the exploration, identification, and definition of medicinal species, which will address taxonomic issues on a species or even interspecific level and also allow us to assess the genetic diversity and population structures within the *Grewioideae* lineage.

Moreover, mVISTA analysis confirmed that the variability in the IR regions was markedly lower than that in the LSC and SSC regions and that protein-coding genes were more conserved compared with noncoding regions. Gene variations were detected in ycf1, psbA, rps16, accD, psaI, psbH, ndhH, ndhF, and accD. Data from chloroplast genomes are essential for species analysis, as organelle-based “barcodes” may be established and then used to assess phylogenetic relationships [53,61]. Several of these loci have previously been utilized as barcode markers in taxonomic identification. These include trnH-psbA, rbcL-accD, ndhF, ycf1, accD, rps16, rps16-trnQ, and petA-psbJ [25]. Therefore, evaluating these genes as novel molecular markers for taxonomic studies in *Grewioideae* is crucial.

The inverted repeat regions are highly conserved in almost all angiosperm chloroplast genomes. However, territorial contraction and expansion in these IR regions is not unusual [56]. The present study revealed identical gene organization comprising the junctions of SC–IR regions of the eight *Grewioideae* chloroplast genomes. In contrast, slight differences were noted in the exact positioning of genes at the SC–IR junctions, particularly for the genes ndhF, ycf1, and trnH, similar to that observed in some angiosperms [62]. Interestingly, *Corchorus capsularis* had the largest IR size in the cp genome of the eight species of *Grewioideae*, which might be related to the expanded ycf2 and ndhF genes in the SSC region. The size of chloroplast genomes, including LSC/SSC lengths, gene counts, and the emergence of pseudogenes, varies among species taxa due to the dilation and compression of the IR regions, as noted in previous studies on whole chloroplast genome sequences of *Pinus*, *Plantago,* and *Passiflora* [63,64,65,66].

Specifically, nucleotide diversity analysis of the *Grewioideae* species revealed that inverted repeat (IR) regions presented fewer variable loci compared with single-copy (SC) regions, including the large single-copy (LSC) region. Moreover, genes with Pi values above 0.03 are mainly localized in the LSC region, which enhances the conservation and stability of the IR region. Chloroplast genomes use a copy-dependent repair mechanism [67], which could explain why the IR region exhibited reduced sequence divergence compared with the SC regions. Additionally, nucleotide diversity analysis revealed significantly increased Pi values (0.04–0.06) for the accD, clpP, psbZ, rpl22, trnS-GCU, trnS-GGA, and trnT-UGU genes. These loci impart considerable genetic variability. Prior molecular investigations have indicated significant biogeographical intricacies among *Grewia* species [1]. Therefore, it is essential to emphasize these areas (genes exhibiting Pi values between 0.04 and 0.06) for the investigation of phylogenetic connections and the evaluation of population genetic frameworks, to elucidate the evolutionary lineage, origins, and migratory patterns of *Grewia* species.

The plastid genome is generally stable, uniparentally transmitted, and less prone to homologous allele recombination [68], making it a model system for examining the evolution of genes and phylogenetic relationships. Complete chloroplast genome sequences also contain a larger and more abundant pool of genetic information and have been confirmed as powerful tools for exploring phylogenetic lines and gene evolution [19].

In this work, a phylogenetic tree was constructed based on chloroplast genome sequences to analyze the phylogenetic relationships among subfamilies of *Malvaceae*. Maximum likelihood (ML) and Bayesian inference revealed a well-resolved phylogenetic backbone of *Grewioideae*. The phylogenetic tree created in this study showed high levels of support for most of the clades in the analysis. The phylogenetic framework of *Malvaceae* can be clearly defined into two major clades: Byttneriina and Malvadendrina. This is supported by bootstrap values and posterior probability values of 100 and 1, respectively. This finding is consistent with recently published research on the family *Malvaceae* using cp information from [19,20].

The clade of the subfamily *Grewioideae* was divided into two main subclades (i.e., the tribes *Apeibeae* and *Grewieae*), which is largely consistent with the classification framework noted in [1]. One clade includes species of section *Apeibeae* (i.e., *Corchorus capsularis* and *Corchorus olitorius*), and the second clade includes species of section *Grewieae* (i.e., *Grewia tembensis, Grewia chungii, Grewia biloba*, *Grewia biloba* var*. Parviflora*, *Microcos paniculata*, and *Colona floribunda*).

In this study, *Grewia tembensis* was placed within the *Grewioideae* subfamily and clustered within the *Grewieae* section. All the members of the genus *Grewia* (*Grewia tembensis, Grewia biloba,* and *Grewia biloba* var*. Parviflora* species) formed one clade, with the exception of *Grewia chungii*, which clustered with *Microcos paniculata*; this species is considered synonymous with *Microcosm chungii* [3].

On the other hand, the phylogenetic trees revealed strong phylogenetic affinity between *Malvoideae* and *Bombacoideae*, supporting the strong phylogenetic relationship of *Brownlowioideae* with *Tilioideae* and *Dombeyoideae*. These findings support previous findings [19,20,69]. However, *Sterculioideae* is the sister group of these clades and *Helicteroideae* at the base of Malvadendrina, which is consistent with most of the results of recent evolutionary studies using cp genome data [19,20].

## 5. Conclusions

This study reported the complete chloroplast genome of *Grewia tembensis* and its comparative analysis with *Grewioideae* species. This study provides insight into the basic structures, conservation, and variability of these reservoirs of genomic sequences. Notably, the genomic configuration of *Grewia* species is highly conserved and relatively uniform despite their wide geographical range in diverse environments across Asia, Africa, and the Arabian Peninsula (Table S9). Inverted repeat (IR) regions exhibit greater conservation than single-copy (SC) regions. An average of at least 30 polymorphic nucleotides were identified across *Grewioideae* species (mainly distributed in large single-copy (LSC) regions), with these sites representing regions of nucleic acid polymorphisms. Moreover, 202–234 simple sequence repeat (SSR) markers, predominantly distributed in the SC regions, were identified in the genomes of *Grewioideae* taxa. The SSRs in the present study, with the identified divergent hotspot regions, may find applications as molecular markers in population genetics and phylogenetic studies and in the development of molecular barcoding markers. The phylogenetic analysis in the present work was based on chloroplast genome information, maximum parsimony (MP), and Bayesian inference of taxa belonging to nine subfamilies within the *Malvaceae* family. The findings supported divided the subfamily *Grewioideae* into two main clades—namely, *Apeibeae* and *Grewieae*—with highly supported bootstrap (BS) values (100%) and Bayesian posterior probabilities (PP = 1). The findings derived from this investigation serve as a significant basis for subsequent research pertaining to *Grewia* L., including species identification, evolutionary relationships, and conservation across the *Grewioideae* subfamily and *Malvaceae* family.

## Figures and Tables

**Table 1:** Features of the *Grewia tembensis* chloroplast genome.

Characteristics	Number
Genome size (bp)	158,040
IRA (bp)	25,471
IRB (bp)	25,471
LSC (bp)	86,956
SSC (bp)	20,142
Total no of genes	130
Total no of unique genes	110
rRNA	3
tRNA	28
Protein-coding genes	79
A%	30.89
T (U)%	31.76
G%	18.51
C%	18.85
GC%	37.36

**Table 2:** Genes containing introns and exons in the chloroplast genome of *Grewia tembensis*.

Gene	Regions	Location Start	Location End	Exon I (bp)	Intron I (bp)	Exon II (bp)	Intron II (bp)	Exon III (bp)
atpF	LSC	37,103	38,520	145	863	410		
clpP	LSC	98,503	99,987	71	894	520		
ndhB	IR	12,388	14,613	775	693	758		
		123,142	125,367	775	693	758		
rpl16	LSC	109,214	110,724	9	1103	399		
rpl2	IR	23,800	25,318	399	589	531		
		112,437	113,955	399	589	531		
rpoC1	LSC	46,878	497,18	432	783	1626		
rps16	LSC	30,619	317,56	40	871	227		
trnA-UGC	IR	4827	5700	37	801	36		
		132,055	132,928	37	801	36		
trnK-UUU	LSC	27,121	29,720	37	2528	35		
trnL-UAA	LSC	75,203	75,845	35	558	50		
ycf3	LSC	69,898	71,931	124	744	230	783	153
petB	LSC	10,2866	104,325	6	812	642		
trnG-UCC	LSC	34,345	35,148	31	713	62		
trnI-GAU	IR	130,963	131,991	32	959	40		
		5764	6792	32	959	40		

## Data Availability

The information delineated in this research is accessible within this article as well as in the Supplementary Materials. The new sequencing dataGrewia *tembensis* has been deposited in the NCBI (https://www.ncbi.nlm.nih.gov/, accessed on 21 June 2025), accession number is PV096988.

## Supplementary Materials

The following supporting information can be downloaded at https://www.mdpi.com/article/10.3390/genes16101124/s1, Table S1: Accession numbers for the chloroplast genomes used in this study for comparison. Table S2: Genes detected in the plastid genome of *G. tembensis*. Table S3: Recognition patterns of codon-anticodon pairs and codon usage in the chloroplast genome of *G. tembensis*. Table S4: Predicted RNA editing sites in the chloroplast genome of *G. tembensis*. Table S5: Results of repetitive sequences of the *G. tembensis* chloroplast genome. Table S6: cpSSRs detected in six chloroplast genomes of the *Grewioideae* subfamily. Table S7: The coding and noncoding regions which showed a high level of variance in chloroplast genomes of the *Grewioideae* subfamily. Table S8: The variable regions we identified showing a Pi (nucleotide diversity) value above 0.03 among the *Grewioideae* taxa. Table S9: Wide geographical distribution of the *Grewioideae* species under this study. Figure S1: The distribution of detected SSRs throughout the large single-copy (LSC), small single-copy (SSC), and inverted repeat (IR) domains of the chloroplast genome of genomes of the eight *Grewioideae* spp.

## Abbreviations

CP	Chloroplast
IR	Inverted repeat
SSC	Small single copy
LSC	Large single copy
SC	Single copy
SSR	Simple sequence repeat
PI	Nucleotide diversity
ML	Maximum likelihood
BI	Bayesian inference analysis
