# Supplementary material for: Comparative Analysis of the Chloroplast Genomes of Grewia tembensis Fresen and Closely Related Species of Grewioideae Hochr: A Phylogenetic and Conservation Perspective
Source: Genes (Basel). 2025 Sep 23;16(10):1124. doi: 10.3390/genes16101124 (PMC12564379; doi:10.3390/genes16101124)
Supplement: Supplementary file 1 [file genes-16-01124-s001.zip › genes-3798014-supplementary.pdf]

Table S1: Accession numbers for the chloroplast genomes used in this study for comparison.

Orders	family	Accession Number	Organism
Malvales malvaceae	Tilioideae	OM908760.1	Tilia cordata
		MT683686.1	Tilia miqueliana
		NC_085570.1	Tilia nobilis
		NC_045284.1	Craigia yunnanensis
		ON086805	Excentrodendron hsienmu
	Dombeyoideae	MN533963	Excentrodendron hsienm
		MH606238.1	Pterospermum kingtungense
		MK962315.1	Pterospermum kingtungense
		NC_057978	Pterospermum menglunense
		MN533971	Pterospermum truncatolobatum
	Brownlowioideae	PP419969	*Brownlowia argentata*
		PP419968	*Brownlowia tersa*
		NC_065808	*Diplodiscus trichospermus*
		OP572286	*Diplodiscus trichospermus*
	Sterculioideae	NC_071829.1	*Brachychiton acerifolius*
		NC_036395	*Firmiana pulcherrima*
		NC_037784.1	*Heritiera angustata*
		NC_037784	*Sterculia monosperma*
		NC_063575.1	*Sterculia nobilis*
	Malvoideae	NC_053355.1	*Abelmoschus moschatus*
		GU907100.1	*Gossypium thurberi*
		NC_045873.1	*Hibiscus cannabinus*
		NC_059767	*Malva verticillata*
	Bombacoideae	NC_054162.1	*Bombax buonopozense*
		NC_037494.1	*Bombax ceiba*
		NC_057077.1	*Ceiba speciosa*
		NC_084136.1	*Ochroma pyramidale*
		NC_057439.1	*Pachira macrocarpa*
	Helicteroideae	NC_073110.1	*Durio dulcis*
		PP668207.1	*Durio lowianus*
		PP668204.1	*Durio testudinarius*
		NC_036829.1	*Durio zibethinus*
		NC_085352.1	*Helicteres hirsuta*
		MZ242236.1	*Reevesia rotundifolia*
	Byttnerioideae	PQ359436.1	*Abroma augustum*
		NC_081971.1	*Melochia corchorifolia*
		OQ557154.1	*Theobroma bicolor*
		MZ725366	*Theobroma cacao*
		NC_054233.1	*Theobroma grandiflorum*
	Grewioideae_Grewieae	NC_054164.1	*Colona floribunda*
		ON882041.1	*Grewia biloba var. parviflora*
		NC_058214.1	*Grewia biloba*
		NC_054166.1	*Grewia chungii*

		PP003957.1	*Microcos paniculata*
	Grewioideae_Apeibeae	NC_044467.1	*Corchorus capsularis*
		NC_044468.1	*Corchorus olitorius*
Malvales outgroup	*Bixaceae*	MH751592.1	*Bixa orellana*
	Sphaerosepalaceae	NC_060629.1	*Dialyceras coriaceum*
	Thymelaeaceae	NC_052860.1	*Gonystylus affinis*

Table S2: Genes detected in the plastid genome of *G. tembensis*.

Category	Group of genes	Name of genes
RNA genes	Ribosomal RNA genes (rRNA)	rrn4.5S, rrn16S[a], rrn5S[a], rrn23S[a]
	Transfer RNA genes (tRNA)	trnR-UCU, trnC-GCA, trnT-GGU, trnG-GCC, trnS-GGA, trnF-GAA, trnM-CAU[a], trnV-GAC[a], trnR-ACGa, trnL-UAG, trnN-GUU[a], trnL-CAA[a], trnH-GUG, trnP-UGG, trnW-CCA, trnT-UGU, trnS-UGA, trnE-UUC, trnY-GUA, trnD-GUC,

		trnS-GCU, trnQ-UUG, trnA-UGC(a+), trnI-CAUa, trnL-UAA+, trnI-GAU[+],trnK-UUU+,trnG-UCC+
Ribosomal proteins	Small subunit of ribosome	rps11, rps12(a+), rps14, rps15, rps16+, rps18, rps19, rps2, rps3, rps4, rps7[a], rps8, rps18
Transcription	Large subunit of ribosome	rpl14, rpl16+, rpl2a+, rpl20, rpl22, rpl23[a], rpl32, rpl33, rpl36
	DNA dependent RNA polymerase	rpoA, rpoB, rpoC2, rpoC1[+]
Protein genes	Photosystem I	psaA, psaB, psaC, psaI, psaJ
	Photosystem II	psbA, psbB, psbC ,psbD, psbE, psbF, psbH, psbI, psbJ, psbK, psbM, psbT, psbZ, psbN
	Subunit of cytochrome	petA, petB[+], petD, petG, petL, petN
	Subunit of synthase	atpA, atpB, atpE, atpF[+], atpH, atpI
	Large subunit of RUBISCO	rbcL
	NADH-dehydrogenase	ndhA, ndhB(a+), ndhC, ndhD, ndhE, ndhF, ndhG, ndhH, ndhI, ndhJ, ndhK
Other genes	Maturase	matK
	Subunit acetyl-coA carboxylase	accD
	C-type cytochrome synthesis	ccsA
	Hypothetical proteins	ycf2a, ycf3++, ycf15[a],ycf4
	Component of TIC complex	ycf1
	Protease	clpPa+
	Envelop membrane protein	cemA

+ Gene with one intron, ++ Gene with two intron and a Gene with copie.

Table S3: Recognition patterns of codon–anticodon pairs and codon usage in the chloroplast genome of *G. tembensis*.

Codon	Amino acid	RSCU	tRNA	Codon	Amino acid	RSCU	tRNA
Phe	UUU	1.29	trnF-GAA	Ser	UCU	1.72	
	UUC	0.71			UCC	0.99	trnS-GGA
Leu	UUA	1.81	trnL-UAA		UCA	1.2	trnS-UGA
	UUG	1.3	trnL-CAA		UCG	0.56	

Tyr	UAU	1.61		Cys	UGU	1.5	
	UAC	0.39	trnY-GUA		UGC	0.5	trnC-GCA
ter	UAA	0		ter	UGA	0	
ter	UAG	0		Trp	UGG	1	trnW-CCA
Leu	CUU	1.27		Pro	CCU	1.54	
	CUC	0.4			CCC	0.72	
	CUA	0.81	trnL-UAG		CCA	1.12	trnP-UGG
	CUG	0.4			CCG	0.62	
His	CAU	1.48		Arg	CGU	1.27	trnR-ACG
	CAC	0.52	trnH-GUG		CGC	0.46	
Gln	CAA	1.51	trnQ-UUG		CGA	1.36	
	CAG	0.49			CGG	0.45	
Ile	AUU	1.45		Thr	ACU	1.54	
	AUC	0.6	trnI-GAU		ACC	0.76	trnT-GGU
	AUA	0.95			ACA	1.22	trnT-UGU
Met	AUG	1	trnM-CAU		ACG	0.49	
Asn	AAU	1.52		Ser	AGU	1.17	
	AAC	0.48	trnN-GUU		AGC	0.36	trnS-GCU
Lys	AAA	1.49	trnK-UUU	Arg	AGA	1.79	trnR-UCU
	AAG	0.51			AGG	0.66	
Val	GUU	1.46		Ala	GCU	1.76	
	GUC	0.46	trnV-GAC		GCC	0.72	
	GUA	1.5			GCA	1.04	trnA-UGC
	GUG	0.58			GCG	0.48	
Asp	GAU	1.6		Gly	GGU	1.26	
	GAC	0.4	trnD-GUC		GGC	0.44	trnG-GCC
Glu	GAA	1.48	trnE-UUC		GGA	1.53	trnG-UCC
	GAG	0.52			GGG	0.76	

Table S4: Predicted RNA editing sites in the chloroplast genome of *G. tembensis*.

Gene	Nucleotide Position	Amino Acid Position	Codon Conversion	Amino Acid Conversion	Score	Position
rps12	221	74	UCA→UUA	S→L	1.2	2
ndhB	586	196	CAU→UAU	H→Y	1.48	1
ycf2	1891	631	CAU→UAU	H→Y	1.48	1

rpl23	20	7	GCA→GUA	A→V	1.04	2
	71	24	UCU→UUU	S→F	1.72	2
	89	30	UCA→UUA	S→L	1.2	2
matK	634	212	CAU→UAU	H→Y	1.48	1
	748	250	CAU→UAU	H→Y	1.48	1
	1291	431	CUU→UUU	L→F	1.27	1
atpA	914	305	UCA→UUA	S→L	1.2	2
	1148	383	UCA→UUA	S→L	1.2	2
atpF	388	130	CAU→UAU	H→Y	1.48	1
rps2	134	45	ACA→AUA	T→I	1.22	2
	248	83	UCA→UUA	S→L	1.2	2
rpoC2	850	284	CCU→UCU	P→S	1.54	1
	2176	726	CAU→UAU	H→Y	1.48	1
	2398	800	CUU→UUU	L→F	1.27	1
	4141	1381	CCA→UCA	P→S	1.12	1
rpoC1	488	163	UCA→UUA	S→L	1.2	2
	41	14	UCA→UUA	S→L	1.2	2
rpoB	338	113	UCU→UUU	S→F	1.72	2
	551	184	UCA→UUA	S→L	1.2	2
	1487	496	GCU→GUU	A→V	1.76	2
	2426	809	UCA→UUA	S→L	1.2	2
rps14	80	27	UCA→UUA	S→L	1.2	2
	149	50	UCA→UUA	S→L	1.2	2
psaB	673	225	CUU→UUU	L→F	1.27	1
rps4	496	166	CUU→UUU	L→F	1.27	1
ndhC	323	108	UCA→UUA	S→L	1.2	1
atpB	403	135	CCU→UCU	P→S	1.54	1
rbcL	434	145	ACU→AUU	T→I	1.54	2
	785	262	GCU→GUU	A→V	1.76	2
accD	350	117	UCU→UUU	S→F	1.72	2
	893	298	CCU→CUU	P→L	1.54	2
	914	305	UCU→UUU	S→F	1.72	2
	1421	474	CCU→CUU	P→L	1.54	2
psaI	83	28	UCU→UUU	S→F	1.72	2
psbJ	59	20	CCU→CUU	P→L	1.54	2
psbF	77	26	UCU→UUU	S→F	1.72	2
petL	5	2	CCU→CUU	P→L	1.54	2
rpl20	203	68	GCA→GUA	A→V	1.04	2
	308	103	UCA→UUA	S→L	1.2	2
clpP	559	187	CAU→UAU	H→Y	1.48	1
psbT	89	30	CCA→CUA	P→L	1.12	2
psbN	29	10	UCU→UUU	S→F	1.72	2
rpoA	779	260	GCU→GUU	A→V	1.76	2
rpl36	83	28	UCC→UUC	S→F	0.99	2
ycf1	4450	1484	CUU→UUU	L→F	1.27	1
	4822	1608	CUU→UUU	L→F	1.27	1
	4837	1613	CCA→UCA	P→S	1.12	1
ndhH	10	4	CCU→UCU	P→S	1.54	1
ndhA	341	114	UCA→UUA	S→L	1.2	2

ndhE	233	78	UCA→UUA	S→L	1.2	2
ndhD	383	128	UCA→UUA	S→L	1.2	2
	878	293	UCA→UUA	S→L	1.2	2
	1298	433	UCA→UUA	S→L	1.2	2
	1310	437	UCA→UUA	S→L	1.2	2
ndhF	262	88	CUU→UUU	L→F	1.27	1
	2194	732	CUU→UUU	L→F	1.27	1

Table S5: Results of repetitive sequences of the *G. tembensis* chloroplast genome.

Repeat Size	Repeat Position 1	Repeat Type	Repeat Size	Repeat Position 2	E-value
75	65943	F	75	68167	4.92E-36

72	137754	P	72	157968	3.15E-34
64	35170	P	64	35170	2.06E-29
48	102233	P	48	102233	8.87E-20
32	33722	P	32	33722	3.81E-10
32	63446	P	32	63446	3.81E-10
30	40475	F	30	40497	6.09E-09
29	33480	P	29	72852	2.44E-08
27	10666	P	27	71107	3.90E-07
27	71107	F	27	127061	3.90E-07
26	78123	F	26	154870	1.56E-06
24	10664	F	24	146859	2.50E-05
24	30040	F	24	30063	2.50E-05
24	127066	P	24	146859	2.50E-05
24	155514	P	24	155514	2.50E-05
23	1543	F	23	1575	9.98E-05
23	1543	P	23	136156	9.98E-05
23	1575	P	23	136188	9.98E-05
23	82758	R	23	82758	9.98E-05
23	136156	F	23	136188	9.98E-05
22	71112	P	22	146861	3.99E-04
22	74593	F	22	74614	3.99E-04
21	33485	F	21	62498	1.60E-03
21	34534	P	21	91869	1.60E-03
21	62498	P	21	72855	1.60E-03
21	151789	R	21	151789	1.60E-03
20	5675	F	20	79777	6.39E-03
20	72091	F	20	109118	6.39E-03
20	76255	P	20	79776	6.39E-03
20	78514	R	20	78514	6.39E-03
20	79777	P	20	132059	6.39E-03
20	96292	R	20	96292	6.39E-03
19	5675	P	19	76255	2.56E-02
19	18057	F	19	18105	2.56E-02
19	18057	P	19	119630	2.56E-02
19	18105	P	19	119678	2.56E-02
19	25493	R	19	63381	2.56E-02
19	30517	R	19	30517	2.56E-02
19	35120	F	19	63798	2.56E-02
19	38080	F	19	38099	2.56E-02
19	40322	C	19	76425	2.56E-02
19	40324	R	19	40324	2.56E-02
19	59319	F	19	63344	2.56E-02
19	63394	P	19	63502	2.56E-02
19	76255	F	19	132060	2.56E-02

19	76895	R	19	76895	2.56E-02
19	106142	R	19	106142	2.56E-02
19	119630	F	19	119678	2.56E-02
19	147319	R	19	147319	2.56E-02

Table 6: cpSSRs detected in six chloroplast genomes of the *Grewioideae* subfamily

**SSR type**	**Repeat unit**	***Grewia tembensis***	***Colona floribunda***	***Corchorus capsularis***	***Corchorus olitorius***	***Grewia biloba***	***Grewia biloba* var.*Parviflora***	***Grewia chungii***	***Microcos paniculata***
Mono	A	94	70	101	92	89	89	90	87
	C	4	5	4	4	4	4	7	5
	G	2	3	4	2	2	2	1	1
	T	89	94	80	89	93	93	93	93
Di	AG/CT	3	1	2	2	3	3	1	1
	AC/GT	2	2	2	2	0	0	2	2
	AT/AT	7	8	13	9	10	10	8	7
Tri	AAG/CTT	0	0	0	0	0	0	0	0
	AAT/ATT	2	6	1	2	2	2	5	4
	ATC/ATG	0	0	4	0	0	0	0	0
Tetra	AAAG/CTTT	1	0	1	1	1	1	1	0
	AAAT/ATTT	6	2	7	7	6	6	3	2
	AAGT/ACTT	0	0	0	2	0	0	0	0
	AATT/AATT	0	1	0	0	0	0	1	1
	AATC/ATTG	1	1	1	1	1	1	1	1
	AAAC/GTTT	0	0	0	1	0	0	0	0
	AGAT/ATCT	0	0	1	0	0	0	0	0
	AATG/ATTC	1	1	0	0	1	1	2	1
	AACT/AGTT	0	1	0	0	0	0	1	0
Penta	AAAAT/ATTTT	1	2	1	0	1	1	1	1
	AATAT/ATATT	1	0	1	2	0	0	0	0
	AAAGT/ACTTT	2	2	0	2	2	2	2	2
	AACAC/GTGTT	2	2	0	0	2	2	2	0
	AAAAG/CTTTT	0	1	1	0	0	0	0	0
	AAGAT/ATCTT	0	0	1	0	0	0	0	0
	AACAT/ATGTT	0	0	1	1	0	0	0	0
	AAATT/AATTT	0	0	2	0	1	1	1	0
	AAACT/AGTTT	0	0	1	1	0	0	0	0
Hexa	AATCAG/ATTCTG	0	0	1	1	0	0	0	0
	AAAAAG/CTTTTT	0	0	1	0	0	0	0	0
	AAAAAT/ATTTTT	0	0	1	0	0	0	0	0
	AAATAT/ATATTT	0	0	2	0	0	0	0	0

Table S7: The coding and noncoding regions which showed a high level of variance in chloroplast genomes of the *Grewioideae* subfamily.

**Regions**	**loci**
Non-coding	rp12-psbA, psbA-trnK-UUU, trnK-UUU-rps16, rps16-trnQ-UUG, psbk-psbi, trnS-trnG-UCC, atpF-atpH, atpH-atpI, rps2-rpoC2, rpoC1-rpoB, rpoB-trnC-GCA, trnC-GCA-petN, petN-psbM, psbM-trnD-GUC, trnD-GUC-trnY-GUA, trnE-UUC-trnT-GGU, trnT-GGU-psbD, psbC-trnS-UGA, trnS-UGA-psbZ, psbZ-trnG-GCC, trnG-GCC-trnM-CAU, trnM-CAU-rps14, rps14-psaB, psaA-ycf3, ycf3-trnS-GGA, rps4-trnT-UGU, trnL-UUU-trnF-GAA, trnF-GAA-ndhJ, ndhC-tmM-CAU, tmM-CAU-atpE, atpB-rbcL, rbcL-accD, accD-psaI, psaI-ysf4, ycf4-cemA, cemA-petA, petA-psbJ, psbE-petL, trnP-UGG-psaJ, psaJ-rpI33, rpI33-rps18, rps18-rpI20, rpI20-rps12, psbH-petB, petB-petD, petD-rpoA, ndhH-rps8, rps8-rpI14, rpI14-rpI16, rpI16-rps3, rps3-rpI22, rpI22-rps19, ycf2, ysf2-ysf15, trnL-CAA-ndhB, rpoB-trnC-GCA, rps7-trnV-GAC, trnA-UGC-rm23S, rm23S-rrn5S, trnR-ACG-trnN-GUU, trnN-GUU-ycf1, ysf1-rps15, ndhA-ndhI, ndhI-ndhG, ndhG-ndhE, ndhE-psaC, rpoB-trnC-GCA, ndhD-ccsA, ccsA-trnL-UAG, trnL-UAG-rpI32, and rpI32-ndhF.
Coding	atpF, rpoC2, accD, clpP, ycf2, ysf1, and rm23S.

Table S8: The variable regions we identified showing a Pi (nucleotide diversity) value above 0.03 among the *Grewioideae* taxa.

Gene	Median_Midpoint	Median_Pi	Closest_Midpoint	Closest_Pi	Region
accD	85980	0.06083	85980	0.06083	LSC
atpA	35636	0.03857	35636	0.03857	LSC
atpH	39294	0.03048	39294	0.03048	LSC
clpP	99206	0.05149	99206	0.05149	LSC
ndhJ	77039	0.03095	77039	0.03095	LSC
petA	91216	0.03131	91216	0.03131	LSC
psaI	87466	0.05077	87466	0.05077	LSC
psaJ	95400	0.03595	95400	0.03595	LSC
psbA	25931	0.03673	25931	0.03673	LSC
psbZ	63101	0.04583	63101	0.04583	LSC
rbcL	84381	0.03435	84381	0.03435	LSC
rpl16	110343	0.03494	110343	0.03494	LSC
rpl20	97212	0.03533	97112	0.03524	LSC
rpl22	111853	0.04527	111753	0.04548	LSC
rpl33	96039	0.03571	96039	0.03571	LSC
rpl36	107601	0.03119	107601	0.03119	LSC
rpoC2	43900	0.03113	43900	0.03113	LSC
rps14	64210	0.03887	64210	0.03887	LSC
rps16	31429	0.03869	31429	0.03869	LSC
rps18	96605	0.034555	96505	0.03458	LSC
rps19	112183	0.03339	112183	0.03339	LSC
rps3	111153	0.034525	111153	0.03458	LSC
rps8	108306	0.03161	108306	0.03161	LSC
trnE-UUC	57619	0.03399	57619	0.03399	LSC
trnK-UUU	28561	0.03345	28561	0.03345	LSC
trnL-UAA	75549.5	0.03738	75560	0.03738	LSC
trnS-GCU	33496	0.04024	33496	0.04024	LSC
trnS-GGA	72814	0.04214	72814	0.04214	LSC
trnT-UGU	74248	0.06089	74248	0.06089	LSC
ycf3	70081	0.03208	70081	0.03208	LSC

Table S9: Wide geographical distribution of the *Grewioideae* species under this study.

Grewioideae species	Distribution	References
Grewia tembensis	The native range of this species is Algeria, NE. Sudan to N. Somalia, SW. Arabian Peninsula. It is a shrub and grows primarily in the desert or dry shrubland biome.	Royal Botanic Gardens, (Kew)& Plants of the World Online [3]
Grewia biloba var. *Parviflora*	The native range of this variety is Central & S. China. It is a shrub or tree and grows primarily in the temperate biome.	
Grewia biloba	The native range of this species is Central & S. China to Korea, Taiwan. It is a shrub or tree and grows primarily in the temperate biome.	
Grewia chungii	The native range of this species is China (S. Yunnan) to Vietnam, Hainan. It is a tree and grows primarily in the wet tropical biome.	
Microcos paniculata	The native range of this species is NE. Pakistan to S. China and Malesia. It is a shrub or tree and grows primarily in the wet tropical biome.	
Corchorus capsularis	The native range of this species is Indian Subcontinent to Central & S. China. It is annual and grows primarily in the subtropical biome.	
Corchorus olitorius	The native range of this species is the Tropical & Subtropical Old World. It is an annual and grows primarily in the seasonally dry tropical biome.	
Colona floribunda	The native range of this species is Assam to China (S. Yunnan) and Indochina. It is a tree and grows primarily in the subtropical biome.	

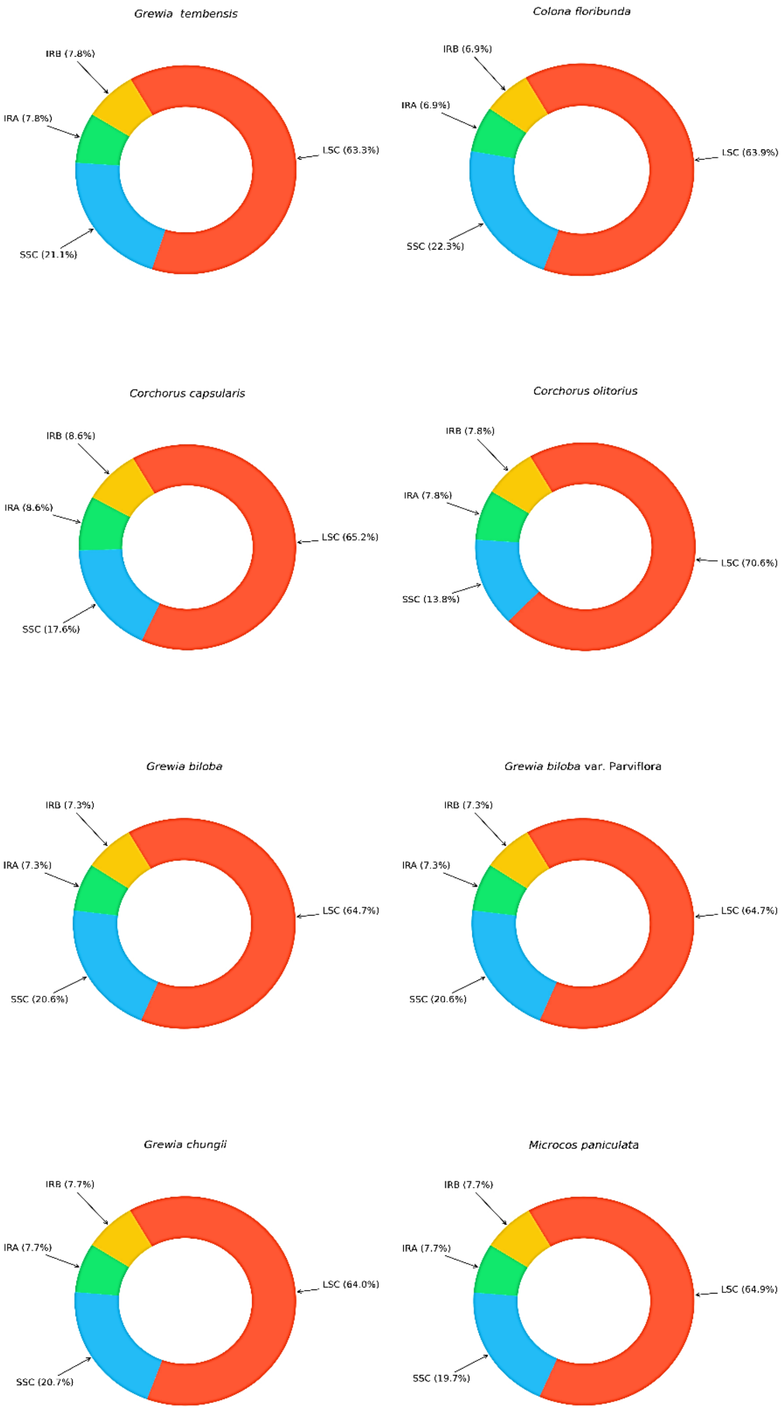

Figure S1: The distribution of detected SSRs throughout the large single-copy (LSC), small single-copy (SSC), and inverted repeat (IR) domains of the chloroplast genome of genomes of the eight *Grewioideae* spp.
